# Octupole-driven magnetoresistance in an antiferromagnetic tunnel junction

**DOI:** 10.1038/s41586-022-05463-w

**Published:** 2023-01-18

**Authors:** Xianzhe Chen, Tomoya Higo, Katsuhiro Tanaka, Takuya Nomoto, Hanshen Tsai, Hiroshi Idzuchi, Masanobu Shiga, Shoya Sakamoto, Ryoya Ando, Hidetoshi Kosaki, Takumi Matsuo, Daisuke Nishio-Hamane, Ryotaro Arita, Shinji Miwa, Satoru Nakatsuji

**Affiliations:** 1 Department of Physics, University of Tokyo, Tokyo, Japan; 2 Institute for Solid State Physics, University of Tokyo, Chiba, Japan; 3 CREST, Japan Science and Technology Agency, Saitama, Japan; 4 Research Center for Advanced Science and Technology, University of Tokyo, Tokyo, Japan; 5 PRESTO, Japan Science and Technology Agency, Saitama, Japan; 6 RIKEN, Center for Emergent Matter Science, Saitama, Japan; 7 Trans-scale Quantum Science Institute, University of Tokyo, Tokyo, Japan; 8 Canadian Institute for Advanced Research, Toronto, Ontario Canada

**Keywords:** Spintronics, Topological insulators, Information storage, Magnetic properties and materials

## Abstract

The tunnelling electric current passing through a magnetic tunnel junction (MTJ) is strongly dependent on the relative orientation of magnetizations in ferromagnetic electrodes sandwiching an insulating barrier, rendering efficient readout of spintronics devices^1–5^. Thus, tunnelling magnetoresistance (TMR) is considered to be proportional to spin polarization at the interface^1^ and, to date, has been studied primarily in ferromagnets. Here we report observation of TMR in an all-antiferromagnetic tunnel junction consisting of Mn_3_Sn/MgO/Mn_3_Sn (ref. ^6^). We measured a TMR ratio of around 2% at room temperature, which arises between the parallel and antiparallel configurations of the cluster magnetic octupoles in the chiral antiferromagnetic state. Moreover, we carried out measurements using a Fe/MgO/Mn_3_Sn MTJ and show that the sign and direction of anisotropic longitudinal spin-polarized current in the antiferromagnet^7^ can be controlled by octupole direction. Strikingly, the TMR ratio (about 2%) of the all-antiferromagnetic MTJ is much larger than that estimated using the observed spin polarization. Theoretically, we found that the chiral antiferromagnetic MTJ may produce a substantially large TMR ratio as a result of the time-reversal, symmetry-breaking polarization characteristic of cluster magnetic octupoles. Our work lays the foundation for the development of ultrafast and efficient spintronic devices using antiferromagnets^8–10^.

## Abstract (web summary)

The authors report observation of tunnelling magnetoresistance in an all-antiferromagnetic tunnel junction consisting of Mn_3_Sn/MgO/Mn_3_Sn, laying the foundation for the development of ultrafast and efficient spintronic devices using antiferromagnets.

## Main

The discovery of giant magnetoresistance^11^ and tunnelling magnetoresistance (TMR)^1–5^ has facilitated rapid progress in the highly integrated and efficient spintronic technologies based on ferromagnets. TMR and spin-transfer torque (STT), due to longitudinal spin-polarized current, provide the reading and writing protocols for the two-terminal magnetoresistive random access memory (MRAM) recently commercialized^12–14^. In addition to the well-established ferromagnetic spintronics, antiferromagnets have attracted tremendous interest^6–10,15^ as next-generation active elements for further improvement in operation speed and integration density. Whereas no STT method has been demonstrated experimentally, various writing means have been developed^16–19^, including spin-orbit torque^20–22^, using the same methods used for ferromagnets. In regard to readout, the anomalous Hall effect^6,23–26^ has recently become available for antiferromagnets in addition to anisotropic magnetoresistance^8–10,18,27^ and spin Hall magnetoresistance^19^. In addition, tunnelling anisotropic magnetoresistance (TAMR) has been investigated for junctions based on a single antiferromagnetic electrode^28–30^. However, TAMR is generally seen at low temperatures and those much lower than the TMR, even when it becomes available at room temperature.

Regarding the perspective of future applications, it is highly important to develop TMR using all-antiferromagnetic MTJs, which potentially generates a high magnetoresistance ratio. On the other hand, research and development of the TMR effect has been restricted primarily to ferromagnetic MTJs because the effect is considered to derive from finite spin polarization at the interface between the magnetic electrodes and insulating barrier^1–5^. Thus, no reports have been published to date on the TMR effect using an all-antiferromagnetic MTJ. In addition, as the basis for designing the STT–MRAM based solely on antiferromagnets, observation and manipulation of longitudinal spin-polarized current is necessary but, again, has never been performed for antiferromagnets. Here we report our discovery of a finite TMR of approximately 2% using a MTJ solely comprising an antiferromagnet. Moreover, our experiment has further clarified the existence of the anisotropic, longitudinal spin-polarized current in an antiferromagnet. Strikingly, the size of TMR in the all-antiferromagnetic MTJ is much larger than the conventional estimate based on spin polarization and thus should derive from a new mechanism.

**Fig. 1:**
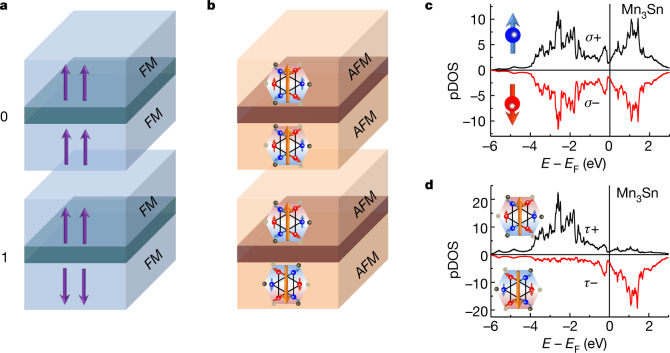
Antiferromagnetic tunnel junction with cluster magnetic octupoles. **a**, Schematics of ferromagnetic (FM) tunnel junction sandwiching an insulating barrier (middle layer, darker colour); the parallel 0 and antiparallel 1 configurations of ferromagnetic moments (purple arrows) generate two different resistance states. **b**, Schematics of a tunnel junction made solely of antiferromagnetic (AFM) electrodes with a magnetic order parameter-breaking TRS, such as the cluster magnetic octupole in Mn_3_Sn. The antiferromagnet Mn_3_Sn has a hexagonal Ni_3_Sn-type structure (space group P63/mmc)^6^, and Mn moments (red and blue arrows) form a cluster magnetic octupole (orange arrows) consisting of six spins on the kagome bilayer. States 0 and 1 arise when the top and bottom octupole polarizations are parallel and antiparallel, respectively. **c**, Projected density of states (pDOS) onto 3*d* orbitals computed using DFT for two opposite spin states with *σ*^+^ and *σ*^−^ in Mn_3_Sn. The pDOS is symmetric in terms of spin polarization (red and blue arrows). Here the direction of spin polarization is parallel to the *a* axis of the crystal. **d**, Calculated pDOS with projection onto octupolar ordered states with opposite octupole moments *τ*+ and *τ*−. Here the polarization direction of the cluster magnetic octupole is parallel to the *a* axis of the crystal and lies in the kagome plane. Inset, corresponding octupole-majority and -minority states used for calculations. Black and red lines correspond to octupole-majority (pDOS_maj_) and octupole-minority (pDOS_min_) DOSs, respectively. The absolute value of pDOS_maj_ is higher when energy level *E* is lower than Fermi energy *E*_F_, whereas that of pDOS_min_ dominates at higher *E*. The polarity of octupole polarization changes sign by tuning the Fermi energy of Mn_3_Sn (Extended Data Fig. 2c). Source data

Ferromagnetic TMR produces the binary signals '0' and '1', respectively, via parallel and antiparallel arrangements of spin polarization in a pair of ferromagnetic electrodes (Fig. 1a). In principle, analogous binary states can be defined for an antiferromagnetic MTJ providing that the antiferromagnetic states break time-reversal symmetry (TRS) macroscopically. Such antiferromagnets have recently been discovered and intensively studied for the development of antiferromagnetic spintronics, because they produce large transverse effects despite vanishingly small magnetization^6,23–26,31,32^. Breaking of TRS in these antiferromagnetic states leads to characteristic electronic structures such as magnetic Weyl semimetal state^33,34^, spin-splitting bands^7,31,32,35,36^ and polarization of cluster magnetic multipoles^37^.

Here we focus on a specific example, the chiral antiferromagnet Mn_3_Sn. This hexagonal kagome metal has attracted much interest because it exhibits large transverse responses normally absent in antiferromagnets^6,38–40^. Below the Néel temperature *T*_N_ ≈ 430 K, all Mn moments of approximately 3 μ_B_ lying in the kagome plane form an antichiral 120° spin order. The magnetic texture can be viewed as a ferroic order of cluster magnetic octupole lying in the kagome plane (Fig. 1b and Methods)^37,39^. This ferroic octupole order breaks the TRS macroscopically and allows us to define states 0 and 1 as the parallel and antiparallel configurations, respectively, of octupole polarization (Fig. 1b, orange arrows and Methods). In addition, it is ferroic octupole order that stabilizes the magnetic Weyl semimetal state^33,34^ and drives large transverse responses such as the anomalous Hall effect^6^, anomalous Nernst effect^38^ and magneto-optical Kerr effect (MOKE)^39^.

To further demonstrate the analogy between spin polarization in ferromagnets, and octupole polarization in antiferromagnets, we drew the projected density of states (DOS) for the positive and negative polarization of spins (Fig. 1c) and magnetic octupoles (Fig. 1d) in the chiral antiferromagnetic state of Mn_3_Sn. In sharp contrast with the symmetric DOS projected onto spin-up and -down states (Fig. 1c), we found a clear energy shift between the majority and minority bands of the octupole moment (Fig. 1d and Methods), closely resembling the shift in the majority and minority bands of the spin moment in ferromagnetic iron (Extended Data Fig. 2a). For conventional ferromagnetic TMR, the imbalance in spin polarization is the key origin according to the basic model for TMR proposed by Julliere^1^ (Fig. 1a, Extended Data Fig. 2a,b and Methods). This suggests that similar TRS breaking due to the imbalance seen in octupole polarization might lead to antiferromagnetic TMR (Fig. 1b,d).

To examine whether a finite TMR effect can be generated between the parallel and antiparallel arrangements of the cluster magnetic octupole moments in Mn_3_Sn, we carried out a theoretical simulation from first principles (Methods and Extended Data Fig. 3). We used specific geometry for calculations—namely, the Mn_3_Sn/vacuum/Mn_3_Sn (0001) structure, in which octupole moments are inside the plane perpendicular to the conducting path. We calculated tunnelling conductances for the parallel and antiparallel arrangements of the octupole moments at the Fermi level, which give the finite positive TMR ratio (Extended Data Fig. 3d and Methods). Although the net magnetic moments of Mn_3_Sn were vanishingly small, the calculated TMR ratio was as large as that obtained in a ferromagnetic counterpart, Fe/vacuum/Fe MTJ (Extended Data Fig. 3i). This indicates that Mn_3_Sn has great potential for use in TMR-based devices such as MRAM, because of the macroscopic TRS breaking captured by cluster octupole moments (Methods). In addition, we found that the transmission properties are robust against both interfacial disorder and the relative lateral shift of the atomic layer at the interface, being useful for TMR applications (Methods, Extended Data Fig. 5 and Supplementary Figs. 4 and 5).

**Fig. 2:**
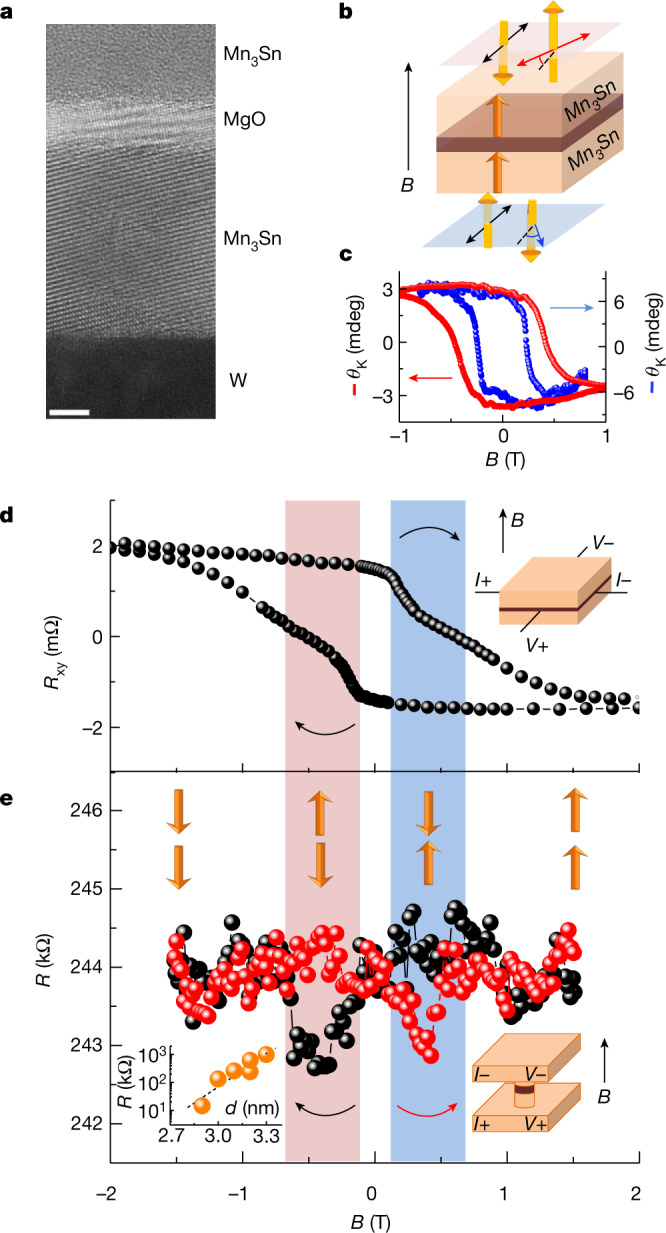
Observation of room-temperature magnetoresistance in the antiferromagnetic tunnel junction based on the chiral antiferromagnet Mn_3_Sn. **a**, High-resolution transmission electron microscopy image of the heterostructure consisting of the antiferromagnetic tunnel junction, W (9 nm)/Mn_3_Sn (12 nm)/MgO (3.3 nm)/Mn_3_Sn (42 nm)/Ta (5 nm), on the MgO substrate. Scale bar, 3 nm. Continuous layers and sharp interfaces were observed (Extended Data Figs. 9 and 10). **b**, Schematic illustration of MOKE measurements. Net polarization directions of magnetic octupoles are denoted by orange arrows. A polarized light beam was applied perpendicular to the film plane (from both the bottom and top), and reflected light became elliptically polarized with the major axis rotated by polar MOKE angle *θ*_K_. **c**, Field dependence of *θ*_K_ for polarized light applied to the top plane (red) and bottom plane (blue) of Mn_3_Sn. **d**, Hall resistance versus magnetic field, *B* applied perpendicular to Mn_3_Sn planes and electric current, *I*. **e**. Field dependence of tunnelling resistance measured in magnetic field, *B* and electric current, *I*, both applied perpendicular to the Mn_3_Sn plane. Four different octupole configurations were obtained, namely from the positive high-field side and up–up, down–up (blue region), up–down (red region) and down–down configurations. Here we show only octupole configurations, for visual clarity. Inset, tunnelling resistance *R* as a function of MgO thickness *d*. **d**,**e**, Schematics for measurement configurations are also shown in insets (Methods). The TMR sign is negative (Methods). In addition, our measurements of the inelastic electron tunnelling spectrum suggest that magnon-electron scattering in the energy range up to 0.1 eV may play a key role in TMR (Supplementary Information and Supplementary Fig. 3). Source data

Motivated by this possibility, we fabricated the Mn_3_Sn-based MTJ to demonstrate the TMR effect corresponding to binary states 0 and 1. To observe reliable tunnelling conduction, preparation of a continuous and smooth tunnelling barrier is essential. As shown in Fig. 2a, we fabricated the stack consisting of W (9 nm)/Mn_3_Sn (12 nm)/MgO (3.3 nm)/Mn_3_Sn (42 nm)/Ta (5 nm) from the MgO substrate side (Methods). According to theory, tunnelling conductance can be potentially anisotropic (Supplementary Information and Supplementary Fig. 1). On the other hand, characterization of films by Hall and Kerr effects may be possible only when octupole polarization has the out-of-plane component. Given these two constraints, we fabricated an epitaxial film of Mn_3_Sn with the $(01\bar{1}1)$ orientation aligned close to the out-of-plane direction by taking advantage of the fact that the MgO barrier tends to grow along the [001] direction (Extended Data Fig. 10 and Methods). Transmission electron microscopy (TEM) imaging confirmed the presence of stack films with a sharp and continuous interface between Mn_3_Sn and MgO (Fig. 2a, Extended Data Fig. 9 and Methods). In addition, the *c* axis of Mn_3_Sn was found to be approximately 30° off the normal direction of the film (Extended Data Fig. 10 and Methods), allowing us to characterize the films and to measure tunnelling conduction in the Mn_3_Sn/MgO/Mn_3_Sn trilayer, as discussed below.

For observation of the TMR effect, the top and bottom Mn_3_Sn electrodes are required to have two distinctive coercivities so that the parallel and antiparallel configurations of octupole polarizations arise as a function of magnetic field (Fig. 1b). We characterized the coercivities of the two Mn_3_Sn layers in the MTJ by field-sweep measurements of polar MOKE at room temperature, where a polarized light beam was injected into both top and bottom layers (Fig. 2b and Methods). Figure 2c presents polar MOKE loops measured in magnetic field *B* applied perpendicular to the plane. The top and bottom Mn_3_Sn layers produced clear hysteresis, with square loops as a function of field *B,* and yielded sizeable changes in Kerr rotation angles ∣*θ*_K_∣ (Fig. 2c). It should be noted that the coercive field of *B*_c_, at approximately 0.5 T for the top Mn_3_Sn (Fig. 2c, red), is twofold that of *B*_c _at 0.25 T for the bottom layer (Fig. 2c, blue). Therefore, these two coercivities should generate state 1 when top and bottom octupole moments are antiparallel.

We further examined the formation of binary states via Hall measurements in a magnetic field perpendicular to the film plane. To stabilize state 0, in which octupole moments in both the top and bottom Mn_3_Sn point upward, we first applied the magnetic field of +2 T, which is larger than all coercive fields. By sweeping the field from +2 to −2 T (Fig. 2d) we found two transitions at *B*_c_ (approximately −0.2 and approximately −0.6 T) close to the coercivities seen in the MOKE measurements above, supporting the premise that coercive fields are nearly identical in the bulk and interface detected by the Hall effect and MOKE measurements, respectively. When the field passed through the smaller coercivity at *B*_c_ (approximately –0.2 T), the octupole moment in the bottom Mn_3_Sn layer reversed, producing state 1 with antiparallel configuration (red regions in Fig. 2d,e). When the field passed through the larger coercivity of *B*_c_(approximately −0.6 T), the octupole moment in the top layer also began rotating downwards, establishing state 0 in which octupole moments were again parallel. When the field was scanned back from −2 to +2 T, these two transitions also occurred at *B*_c_(approximately 0.2 and approximately 0.6 T), stabilizing a further state 1 between 0.2 and 0.6 T (blue regions in Fig. 2d,e), as well as a state 0 greater than 0.6 T.

We then moved our focus to the field dependence of the TMR in the antiferromagnetic MTJ. By scanning the field applied perpendicularly to the film plane from +1.5 to −1.5 T, we found that resistance exhibited a clear drop when *B* was between −0.2 and −0.6 T. This low-resistance state (LRS) appeared again between 0.2 and 0.6 T when we swept the field backwards from −1.5 to +1.5 T. The LRS and the remaining high-resistance state (HRS) correspond to states 1 and 0 in the MOKE and Hall measurements (Fig. 2c,d)—that is, the antiparallel and parallel configurations of the top and bottom octupole moments, respectively. By adapting the definition TMR = (HRS – LRS)/LRS × 100%, we estimated the TMR ratio to be around 0.6% at room temperature in this device. The inset in Fig. 2e shows tunnelling resistance as a function of MgO thickness; the exponential increase in resistance confirmed the reliability of tunnelling conduction.

Our first observation of TMR in the all-antiferromagnetic tunnel junction Mn_3_Sn/MgO/Mn_3_Sn is outstanding. On the other hand, the magnitude is still small, raising the question of whether this arose from the TRS-breaking octupole polarization—as our theory suggests—or from spin polarization, which is widely accepted to generate the conventional TMR effects^1–5^. Previous theoretical work indicates that Mn_3_Sn may host the longitudinal spin-polarized current^7^ because the non-collinear antiferromagnetic state breaks the TRS macroscopically^6,7^. In the following, we examine the latter scenario by investigating spin-polarized current in Mn_3_Sn through the conventional TMR scheme^1^.

**Fig. 3:**
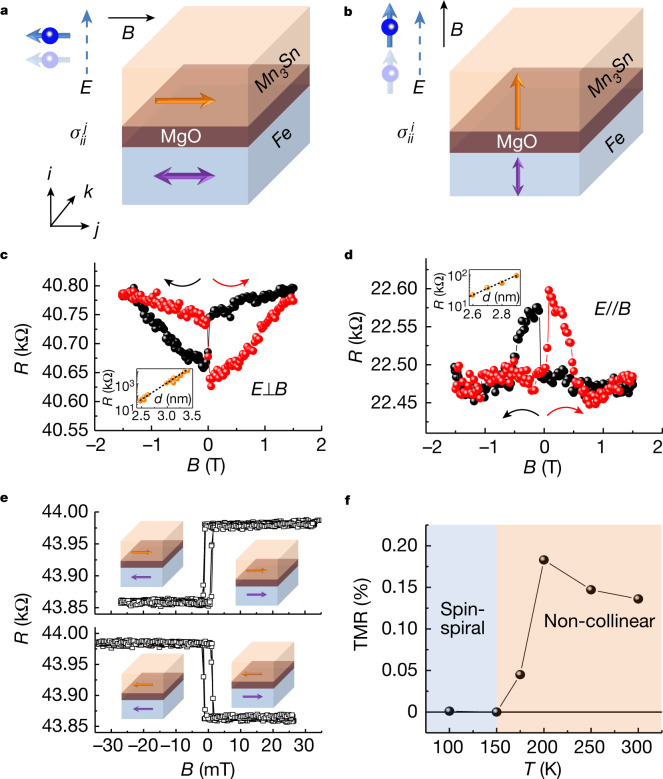
Anisotropic longitudinal spin-polarized current induced by electric current from Mn_3_Sn. **a**,**b**, Schematics of the MTJ consisting of Fe/MgO/Mn_3_Sn. The spin current detector Fe has the in-plane easy axis (**a**) and out-of-plane easy axis (**b**) (Methods). The corresponding magnetizations in Fe (purple arrows), net polarization direction of octupole polarization (orange arrows) and spin polarization (small blue arrows) of longitudinal spin current induced along the electric field *E* (dashed blue arrows) in Mn_3_Sn are schematically shown. The schematics also show switching of magnetization in Fe with a small coercivity of approximately a few mT. **c**, Field dependence of TMR measured in magnetic field *B* applied within the Mn_3_Sn film plane, and electric current *I* applied perpendicular to the Mn_3_Sn plane using the MTJ shown in **a**. **d**, Field dependence of TMR measured in magnetic field *B* and electric current *I* applied perpendicular to the Mn_3_Sn film plane using the MTJ shown in **b**. Red (black) curved arrow indicates the direction of the *B* sweep for obtaining TMR data shown in red (black). Insets show tunnelling resistance *R* as a function of MgO thickness *d*. **e**, Magnetic field dependence of TMR measured using the MTJ shown in **a**. The maximum field is 30 mT so that only the Fe moment switches. Results shown in upper and lower panels were obtained after saturating the octupole polarization in Mn_3_Sn with a magnetic field of  +1.5 and −1.5 T, respectively. **f**, Temperature dependence of TMR ratio measured using the MTJ shown in **a**. The TMR exists only when Mn_3_Sn is in the non-collinear antichiral phase (pink region). This behaviour characteristic of Mn_3_Sn indicates that the Mn_3_Sn interfacing MgO and leading to the TMR has properties similar to bulk Mn_3_Sn (Methods). Source data

Theoretically, spin-polarized current is described by a spin-conductivity tensor ${\sigma }_{ij}^{k}$, in which electric field *E* along axis *j* drives the spin current flowing along direction *i* with spin polarization aligned along the *k* axis^7^. The non-collinear antichiral ordering produces momentum-dependent spin polarization on the Fermi surface, and thus the application of an electric field may drive net longitudinal spin-polarized current with the spin-polarization direction along the octupole polarization (Supplementary Fig. 2). To evaluate the magnitude and sign of the longitudinal spin-polarized current generated in Mn_3_Sn, we fabricated a Fe/MgO/Mn_3_Sn MTJ with an in-plane magnetized Fe layer using, respectively, the antiferromagnet Mn_3_Sn and the typical ferromagnet Fe as the source and detector of the spin-polarized current. We can use the magnetic field to orient spin polarization by directing octupole polarization along the magnetic field. First, for detection of ${\sigma }_{ii}^{j}$ we applied the in-plane field so that the directions of electric field *E* and magnetic field *B* (*i* and *j*, respectively) were perpendicular (Fig. 3a). By sweeping the magnetic field along the in-plane direction *j* from +1.5 to −1.5 T and back to +1.5 T, a negative TMR ratio of 0.2% was observed (Fig. 3c). Assuming the spin polarization of Fe/MgO to be around 0.6, analysis based on the Julliere model yields a spin polarization of Mn_3_Sn of about −2 × 10^−3^ (Methods)^1^. Here, the spin-polarization direction (solid blue arrow in Fig. 3a) is perpendicular to the electric field (solid blue arrow) and the negative sign of spin polarization is consistent with the theoretical analysis of ${\sigma }_{ii}^{j}$ (ref. ^7^).

Second, we measured the TMR in another series of Fe/MgO/Mn_3_Sn MTJs with a perpendicular magnetized Fe layer as a function of magnetic field *B* along the out-of-plane direction parallel to electric field *E*, for detection of ${\sigma }_{ii}^{i}$ (Methods and Fig. 3b). In contrast with the in-plane geometry above, a positive ratio of 0.5% (Fig. 3d) was observed in the out-of-plane magnetized Fe/MgO/Mn_3_Sn. Our analysis found the spin polarization of Mn_3_Sn to be approximately +4 × 10^−3^, consistent with the predicted positive sign of ${\sigma }_{ii}^{i}$ for the case in which the spin polarization direction (small blue arrow in Fig. 3b) is parallel to the electric field (blue dashed arrow). The bias dependence of TMR (Extended Data Fig. 6), which may give useful information about the energy dependence of polarization^41^, clarified the symmetric bias dependence of the TMR ratio for the perpendicular (*E*⊥*B*) and parallel (*E*//*B*) configurations (Extended Data Fig. 7), confirming the anisotropy of the longitudinal spin-polarized current in Mn_3_Sn.

To further confirm the TRS-breaking character of the longitudinal spin-polarized current, we examined the TMR in the configuration shown in Fig. 3a after polarizion of the octupoles in Mn_3_Sn with an in-plane magnetic field, *B* of +1.5 T and −1.5 T. For each polarized state we measured the TMR by sweeping the in-plane magnetic field within 30 mT in which only Fe moments were switched. The two different polarities of TMR were clearly observed, corresponding to polarization in +1.5 T (Fig. 3e, top) and −1.5 T (Fig. 3e, bottom), in accordance with the TRS-breaking character of the spin current causing the hysteresis shown in Fig. 3c. Moreover, the TMR in Fe/MgO/Mn_3_Sn exhibited temperature dependence characteristic of Mn_3_Sn (Fig. 3f); it is only in the antichiral non-collinear phase in which the octupoles form a ferroic order that can generate the spin-polarized current^7^. Thus, the observed temperature dependence confirms that the TMR effect in Fe/MgO/Mn_3_Sn is driven by the spin-polarized current generated in Mn_3_Sn.

Finally, the spin polarization experimentally obtained for Mn_3_Sn allowed us to estimate the TMR ratio of Mn_3_Sn/MgO/Mn_3_Sn based on the Julliere model^1^ (Methods). Namely, if we assume that the TMR is based on tunnelling between spin-polarized states as in conventional ferromagnets, the ratio should be about 0.002%, which is more than two orders of magnitude lower than the value of around 0.6% shown in Fig. 2e. In addition, with increased barrier thickness and tunnelling resistance area we found that the ratio was increased up to 1.6% (Extended Data Fig. 8 and Methods). This indicates that the TMR observed in Mn_3_Sn/MgO/Mn_3_Sn originated not from the spin-polarized current or weak ferromagnetic moments due to canting, but from TRS breaking in the antiferromagnet—that is, the momentum-dependent, spin-splitting bands^7,32,35,36^ caused by the polarization of magnetic octupoles^37^.

In conclusion, we have demonstrated that the all-antiferromagnetic tunnel junction Mn_3_Sn/MgO/Mn_3_Sn shows the TMR effect at a ratio of about 2% at room temperature between the parallel and antiparallel alignments of octupole moments. The TMR ratio is far larger than its estimate based on the anisotropic spin polarization found for Mn_3_Sn. Besides, our theory predicts that the antiferromagnetic TMR effect originating from the TRS-breaking magnetic octupole may become as large as conventional TMR commonly seen in ferromagnets. Thus, our first observations of the all-antiferromagnetic TMR effect, as well as the anisotropic spin-polarized current, provide new and useful functionality, opening new directions in the research and development of antiferromagnetic spintronics and magnetic memory technology.

## Methods

### Sample growth

#### Mn_3_Sn/MgO/Mn_3_Sn MTJs

**Extended Data Fig. 1:**
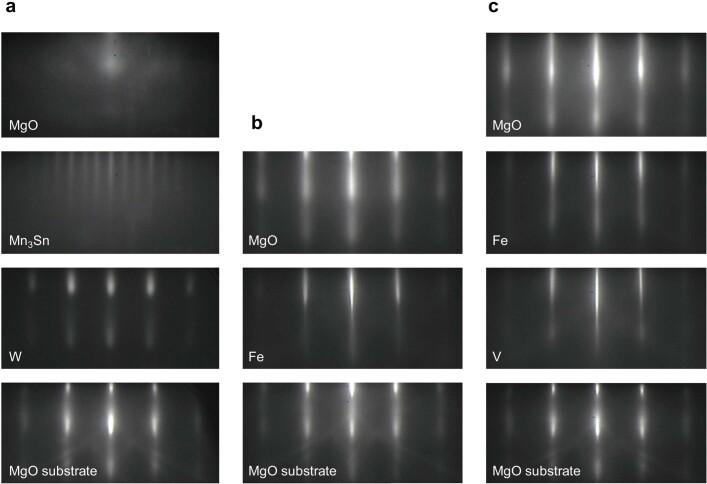
RHEED images in the deposition process of the multilayers. **a**, In-situ reflection high energy electron diffraction (RHEED) images of the MgO (001) substrate after annealing at 800 °C for 10 minutes, the W layer (9 nm) annealed at 800 °C for 10 minutes, the Mn_3_Sn layer (total 12 nm) deposited at 260 °C, and the MgO (3.3 nm) layer deposited at room temperature in the W(9 nm)/Mn_3_Sn(12 nm)/MgO(3.3 nm)/Mn_3_Sn(42 nm)/Ta(5 nm) multilayer. **b**, RHEED images of the MgO (001) substrate after annealing at 800 °C for 10 minutes, the Fe layer (30 nm) annealed at 350 °C for 15 minutes, and the MgO (~ 3 nm) layer deposited at room temperature in the MgO(5 nm)/Fe(30 nm)/MgO(~ 3 nm)/Mn_3_Sn(42 nm)/Ta(5 nm) multilayer. **c**, RHEED images of the MgO (001) substrate after annealing at 800 °C for 10 minutes, the V layer (30 nm) annealed at 500 °C for 20 minutes, the Fe layer (0.6 nm) deposited at room temperature, and the MgO (~ 3 nm) layer deposited at room temperature in the MgO(5 nm)/V(30 nm)/Fe(0.6 nm)/MgO(~ 3 nm)/Mn_3_Sn(42 nm)/Ta(5 nm) multilayer. The incident electron beam was parallel to the MgO [100] direction for all the measurements.

A W (9 nm)/Mn_3_Sn (12 nm)/MgO (3.3 nm)/Mn_3_Sn (42 nm)/Ta (5 nm) (left: substrate side, right: surface side) multilayer was grown on a MgO(001) substrate. The W (9 nm)/Mn_3_Sn (12 nm)/MgO (3.3 nm) layer was fabricated by the molecular beam epitaxy (MBE) method under ultrahigh vacuum (UHV) at a base pressure of 2 × 10^−8^ Pa. The MgO(001) substrate was annealed at 800 °C for 10 min in the MBE chamber before deposition. The W layer (9 nm) was deposited at a rate of 0.1 Å s^−1^ at 300 °C and subsequent annealment at 800 °C for 10 min. The Mn_3_Sn layer (12 nm) was fabricated at a rate of 0.25 Å s^−1^ with coevaporation of Mn and Sn, in which the deposition rate of Mn and Sn was set for the stoichiometric composition Mn_3_Sn. The Mn_3_Sn layer (3 nm) was first deposited at room temperature and then annealed at 320 °C. The extra Mn_3_Sn layer (9 nm) was deposited at approximately 260 °C. Subsequently the MgO layer (3.3 nm) was fabricated at a rate of 0.1 Å s^−1^ at room temperature. The stack was later annealed at 600 °C for 30 min. As shown in Extended Data Fig. 1a, streak patterns were observed by reflection high-energy electron diffraction (RHEED), confirming the formation of epitaxial growth of flat interfaces in the W (9 nm)/Mn_3_Sn (12 nm)/MgO (3.3 nm) layer. The incident electron beam is parallel to the MgO [100] direction. The stack was transferred to a magnetron sputtering chamber with base pressure greater than 5 × 10^−7^ Pa. In the sputtering chamber, the Mn_3_Sn (42 nm)/Ta (5 nm) layer was grown at room temperature by magnetron sputtering at a rate of 0.1 nm s^−1^ and a power of 60 W and Ar gas pressure of 0.5 Pa. After deposition, the entire stack was annealed at 450 °C to crystallize the Mn_3_Sn layer (42 nm), similar to our previous work for polycrystalline Mn_3_Sn^42^.

To investigate crystallinity and orientation, cross-sectional transmission electron microscopy (TEM) images for the W (9 nm)/Mn_3_Sn (12 nm)/MgO (3.3 nm)/Mn_3_Sn (42 nm)/Ta (5 nm) multilayer were taken at room temperature using a commercial TEM system (JEOL, JEM-ARM200F). The maximum operating voltage was 200 kV. Samples for TEM observation were prepared from the TMR device, consisting of the multilayer. using a focused ion beam (Hitachi High-Tech NX2000, Ga 2–30 kV, Ar 1 kV). Before processing, a protective film (C (100 nm)/W (100 nm)) was deposited on an area of 10 × 3 μm^2^ on the sample surface and the sample subsequently thinned by focused Ga and Ar ion beams. The TEM images presented in Fig. 2a and Extended Data Fig. 10a show the sharp interface between the Mn_3_Sn layers and the MgO layer. Nanobeam electron diffraction patterns of the Mn_3_Sn (top), MgO, Mn_3_Sn (bottom) and W layers show epitaxial growth from the W layer to the MgO barrier (Extended Data Fig. 10b–e). As shown in Extended Data Fig. 10a, we fabricated the epitaxial Mn_3_Sn layer on the MgO substrate(001)[010]∣∣W$(001)[\bar{1}10]$ stacks that have the $(01\bar{1}1)$ orientation aligned close to the direction of thickness. In this orientation the kagome plane, which is the magnetic easy plane for the cluster magnetic octupole, is oriented along nearly 60° off the normal direction of the film—that is, the *c* axis of Mn_3_Sn is about 30° off the normal direction of the film, as shown by the transparent green plane in Extended Data Fig. 10f. Here the size of the $(01\bar{1}1)$-oriented Mn_3_Sn crystallite was confirmed to be approximately 100 nm. The MgO barrier on the epitaxial Mn_3_Sn (bottom) layer has around 10 nm crystallites and shows the (001) orientation with a mosaicness of about 10°. An atomic arrangement of each layer shows a potential epitaxial relationship (Extended Data Fig. 10g) in which the lattice constants of MgO, W and Mn_3_Sn are considered to be *a* = 4.21 Å for MgO, *a* = 3.17 Å for W^43^ and *a* = 5.66 Å and *c* = 4.53 Å for Mn_3_Sn^6^. Whereas the Mn arrangement is not perfectly square and may thus have 90°-rotated variants in the film plane, the out-of-plane orientation for all variants should be similar to that observed in the present study (Extended Data Fig. 10a).

#### Fe/MgO/Mn_3_Sn MTJ with an in-plane magnetized Fe layer

A MgO (5 nm)/Fe (30 nm)/MgO (about 3 nm)/Mn_3_Sn (42 nm)/Ta (5 nm) multilayer was deposited on the MgO(001) substrate. The MgO (5 nm)/Fe (30 nm)/MgO (about 3 nm) was fabricated using the MBE method under UHV, as described above for the Mn_3_Sn/MgO/Mn_3_Sn deposition process. The MgO(001) substrate was annealed at 800 °C for 10 min in the MBE chamber before deposition. First, a MgO layer (5 nm) was grown on the substrate at a rate of 0.1 Å s^−1^ at room temperature. Next, the Fe layer (30 nm) was deposited at a rate of 0.25 Å s^−1^ at room temperature and subsequently annealed at 350 °C for 15 min. Finally, the MgO layer (about 3 nm) was fabricated at a rate of 0.1 Å s^−1^ at room temperature. As shown in Extended Data Fig. 1b, clear streak patterns were observed by RHEED, confirming the formation of epitaxial layers with flat interfaces in the MgO (5 nm)/Fe (30 nm)/MgO (about 3 nm) layer. The incident electron beam was parallel to the MgO [100] direction. The stack was transferred to the magnetron sputtering chamber and the Mn_3_Sn (42 nm)/Ta (5 nm) layer additionally grown at room temperature by magnetron sputtering at a rate of 0.1 nm s^−1^ with a power of 60 W and Ar gas pressure 0.5 Pa. After deposition, the entire stack was annealed at 450 °C for 30 min.

#### Fe/MgO/Mn_3_Sn MTJ with a perpendicular magnetized Fe layer

A MgO (5 nm)/V (30 nm)/Fe (0.6 nm)/MgO (about 3 nm)/Mn_3_Sn (42 nm)/Ta (5 nm) multilayer was deposited on the MgO(001) substrate. The MgO (5 nm)/V (30 nm)/Fe (0.6 nm)/MgO (about 3 nm) was fabricated by the MBE method in the UHV MBE chamber. Similar to the method used for the Fe/MgO/Mn_3_Sn stack, we first deposited a MgO layer (5 nm) on the substrate at a rate of 0.1 Å s^−1^ at room temperature. The V layer (30 nm) was deposited at a rate of 0.25 Å s^−1^ at room temperature and subsequently annealed at 500 °C for 20 min. The Fe (0.6 nm) and MgO (about 3 nm) layers were deposited at room temperature at rates of 0.05 and 0.1 Å s^−1^, respectively. As shown in Extended Data Fig. 1c, clear streak patterns were observed by RHEED, confirming the formation of epitaxial layers with flat interfaces in the MgO (5 nm)/V (30 nm)/Fe (0.6 nm)/MgO (about 3 nm) layer. The incident electron beam was parallel to the MgO [100] direction. The stack was transferred to the magnetron sputtering chamber and the Mn_3_Sn (42 nm)/Ta (5 nm) layer additionally grown by magnetron sputtering at room temperature at a rate of 0.1 nm s^−1^ with a power of 60 W and Ar gas pressure 0.5 Pa. After deposition, the entire stack was annealed at 450 °C for 30 min. In this stack the V layer was used as the seed layer, which can help to induce the strong perpendicular magnetic anisotropy of ultrathin Fe (under 1 nm)^44,45^.

We carried out all fabrication processes in situ, including sample transfer from the MBE chamber to the sputtering chamber. The sputtering-grown Mn_3_Sn layer (42 nm) on the MgO layer was used for all samples. The composition of this Mn_3_Sn layer was determined to be Mn_3.15_Sn_0.85_ by scanning electron microscopy–energy-dispersive X-ray spectroscopy. Although this composition is Mn rich, it is also a stable composition for a single phase of the D0_19_ Mn_3_Sn^42^, in which excess Mn randomly occupies the Sn site^6,46^.

### MTJ fabrication and magnetic and transport measurement

We encapsulated MTJ devices in SiO_*x*_, with electrical contacts formed from 60 nm Pt. Hall measurements were conducted at 300 K in a commercial physical property measurement system (Quantum Design). The field dependence of Hall resistivity was obtained after subtracting the longitudinal resistivity contribution, which was found to be constant as a function of magnetic field. Zero-field Hall resistivity, *ρ*_H_(*B* = 0) was estimated as (*ρ*_H_(*B* = +0) − *ρ*_H_(*B* = −0)/2. Here +0 and −0 were used to indicate zero magnetic field approached from +2 and −2 T, respectively. Tunnelling resistance was measured with a two-probe method in a probe system with an electromagnet at room temperature. A commercial source measurement unit (Keithley 2400, Tektronix) was used to measure resistance for the microfabricated magnetic tunnel junctions. Because a fixed resistor of 1 kΩ was series connected with the MTJ to protect it during measurements, MTJ data include 1 kΩ from the fixed resistor. Electrical measurements recorded below room temperature were performed under 10^−4^ Pa in a vacuum chamber cooled by a helium compressor. The sample substrate was fixed on a Cu sample stage and temperature measured by a thermometer inside the sample stage.

### MOKE magnetometry

The magnetic field dependence of MOKE was measured using a commercial system (NanoMOKE3, Quantum Design). The top and bottom Mn_3_Sn layers in the W (9 nm)/Mn_3_Sn (12 nm)/MgO (3.3 nm)/Mn_3_Sn (42 nm)/Ta (5 nm) stacks were used for measurement in a polar MOKE configuration under out-of-plane applied magnetic fields between –1.3 and +1.3 T at room temperature. MOKE loops were acquired using a 660 nm semiconductor laser and a spatial light modulator enabling acquisition of 20 hysteresis loops at a rate of 0.1 Hz. To obtain the MOKE signal, we subtracted the *B*-linear part originating from the extrinsic contribution (for example, a Faraday effect of the optical lenses). Our MOKE measurements for the W (9 nm)/Mn_3_Sn (12 nm)/MgO (3.3 nm)/Mn_3_Sn (42 nm)/Ta (5 nm) stacks showed that the coercive field of *B*_c _(approximately 0.5 T) for the top Mn_3_Sn (Fig. 2c, red) was twice that of *B*_c_ (about 0.25 T) for the bottom layer (Fig. 2c, blue), probably due to the difference in buffer layers facing each Mn_3_Sn (W for bottom and MgO for top), as well as to variation in the thickness of Mn_3_Sn.

### Calculation of the projected density of states

The projected density of states (pDOS) of Mn_3_Sn and body-centred cubic Fe (bcc-Fe) was calculated with the Wannier functions obtained using the WANNIER90 package^47–49^, in which localized Wannier functions are constructed by projection of Bloch wave functions onto atomic orbitals. Bloch wave functions were obtained by density functional theory (DFT) calculations using the QUANTUM ESPRESSO (QE) packages^50,51^. In DFT calculations, the projector-augmented wave pseudopotential with spin-orbit couplings^52^ was used, with exchange correlation taken into account by Perdew–Burke–Ernzerhof-type generalized gradient approximation^53^. For Mn_3_Sn, the lattice constants *a* = 5.665 Å and *c* = 4.531 Å were used^54^ and *k*-point meshes were 7 × 7 × 7 and 8 × 8 × 8 for self-consistent field (scf) and non-scf calculations, respectively. The cut-off energies of wave function and charge density were 80 and 320 Ry, respectively. Bloch wave functions were projected onto the *s*-, *p*- and *d*-orbitals of Mn ions and the *s*- and *p*-orbitals of Sn ions. For bcc-Fe we used the lattice constant *a* = 2.87 Å, and 8 × 8 × 8 and 12 × 12 × 12 *k*-point grids were used for scf and non-scf calculations, respectively. We set the energy cut-offs of wave function and charge density as 80 and 500 Ry, respectively. Bloch states were projected onto the *s*-, *p*- and *d*-orbitals of the Fe ion. In Wannierization we set the *k*-point mesh as 8 × 8 × 8 and 12 × 12 × 12 for Mn_3_Sn and bcc-Fe, respectively. Using Wannier functions we calculated pDOS with the 64 × 64 × 64 *k*-point grid onto cluster magnetic octupolar ordered states of Mn_3_Sn and the magnetic ordered states of bcc-Fe.

### Cluster magnetic octupole and its polarization

For the symmetry operation of the structural D_6h_ point group, the non-collinear magnetic order of Mn_3_Sn has the same transformation properties as the cluster magnetic octupole moment, with ${T}_{\gamma }^{x}=\frac{1}{\sqrt{2}}(-{M}_{31}+{M}_{3-1})$ and ${T}_{\gamma }^{y}=\frac{i}{\sqrt{2}}({M}_{31}+{M}_{3-1})\,$^37,55^. This octupole moment has the same irreducible representation as the ferromagnetic dipole, ${J}_{x}=\frac{1}{\sqrt{2}}(-{M}_{11}+{M}_{1-1})$ and ${J}_{y}=\frac{i}{\sqrt{2}}({M}_{11}+{M}_{1-1})$, and thus its ferroic order breaks time-reversal symmetry macroscopically. This also indicates that the cluster magnetic octupole is parallel to the weak ferromagnetic moment (approximately 7 mμ_B_/Mn) due to spin canting, which arises as a result of the competition between Dzyaloshinskii–Moriya interaction, exchange coupling and single-ion anisotropy^56^. Therefore, the driving mechanism of the anomalous Hall effect of Mn_3_Sn can be interpreted as the ferroic order of the octupole moment of Mn_3_Sn, in the same way as the ferromagnetic order of the dipole moment of Fe. The cluster multipole theory is thus useful for understanding the underlying physics on non-collinear antiferromagnets such as Mn_3_Sn^37^. In the calculation of octupole polarization, we estimated the expectation value of the following operator with *p* = 3 and *q* = ±1 for the Bloch wave functions obtained by generalized gradient approximation calculation:

1 $${\tau }_{pq}^{(\mu )}\equiv \sqrt{\frac{4\pi }{2p+1}}\mathop{\sum }\limits_{i=1}^{{N}_{{\rm{atom}}}^{(\mu )}}{{\boldsymbol{\sigma }}}_{i}\cdot {\nabla }_{i}(| \,{{\bf{R}}}_{i}\,{| }^{p}\,{Y}_{pq}{({\theta }_{i},{\varphi }_{i})}^{* })$$

in which ${N}_{{\rm{atom}}}^{(\mu )}$ is the number of atoms of the *μ*th cluster, ***σ***_*i*_ is the Pauli matrices defined for the spin degrees of freedom of the *i*th atom, ${\nabla }_{i}\equiv \frac{\partial }{\partial {{\bf{R}}}_{i}}$, **R**_*i*_ ≡ (*X*_*i*_, *Y*_*i*_, *Z*_*i*_) is the position of the *i*th atom, *Y*_*pq*_ are the spherical harmonics and **R**_*i*_, *θ*_*i*_ and *ϕ*_*i*_ are the distance, polar angle and azimuthal angle, respectively, of the *i*th atom.

### Estimation of spin polarization of Fe and Mn_3_Sn using the Julliere model

Based on the Julliere model^1^, the relative conduction change, Δ*G* between states 0 and 1 is described as Δ*G* = *G* × 2*P*_1_*P*_2_/(1 + *P*_1_*P*_2_), where *G*, *P*_1_ and *P*_2_ correspond, respectively to the tunnelling conduction and spin-polarization ratio of the effective tunnelling density of states of magnetic electrodes 1 and 2. The spin polarization of Fe/MgO in our device is assumed to be about 0.6 (ref. ^45^).

### Fermi level shift and resultant negative TMR

**Extended Data Fig. 2:**
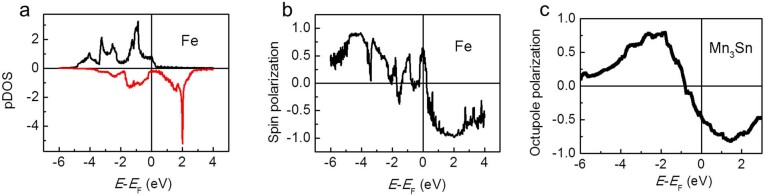
Calculation results of the spin polarization of Fe and the octupole polarization of Mn_3_Sn. **a**, Calculated projected Density of States (pDOS) of Fe for the majority (pDOS_maj_, black) and the minority (pDOS_min_, red) bands with different spin polarizations. **b**, Calculated spin polarization of Fe as a function of the Fermi energy. The spin polarization is normalized by estimating *σ* = (pDOS_maj_ − pDOS_min_)/(pDOS_maj_ + pDOS_min_). **c**, Calculated octupole polarization of Mn_3_Sn as a function of the Fermi energy. The normalized octupole polarization is calculated by *σ* = (pDOS_maj_ − pDOS_min_)/(pDOS_maj_ + pDOS_min_). The polarity of octupole polarization changes the sign when *E* − *E*_F_ is between − 1 and 0 eV.

Given the strong Fermi energy dependence on the sign of octupole polarization (Extended Data Fig. 2c), the sign of TMR should be very sensitive to the Fermi energy of Mn_3_Sn. Therefore, interfacial engineering may influence the Fermi energy of Mn_3_Sn. Extended Data Fig. 4a shows the Hall resistivity of Mn_3_Sn as a function of temperature. When decreasing the temperature, Hall resistivity vanishes due to the phase transition from non-collinear antichiral to spin-spiral. Both the anomalous Hall effect and non-zero spin polarization exist only in the non-collinear antichiral phase of Mn_3_Sn rather than in the spin-spiral phase. Nevertheless, phase transition temperature detected through TMR (black circle) was around 100 K lower than that (blue square) found in Hall resistivity (Extended Data Fig. 4a). This is most probably because the Fermi energy of the interfacial Mn_3_Sn shifted as compared with that of the Mn_3_Sn film, due to its contact with MgO. In addition, the distinct Fermi level shift of Mn_3_Sn at the bottom and top may have arisen due to variation in the thermal annealing process (Methods). Here we roughly simulated normalized TMR via octupole polarizations from bottom *τ*_bottom_ and top Mn_3_Sn *τ*_top_, normalized TMR = *τ*_bottom_ × *τ*_top_, assuming that the difference in Fermi energy shift is 1 eV (Extended Data Fig. 4b).

### First-principles calculation of the tunnelling magnetoresistance effect with Mn_3_Sn electrodes

**Extended Data Fig. 3:**
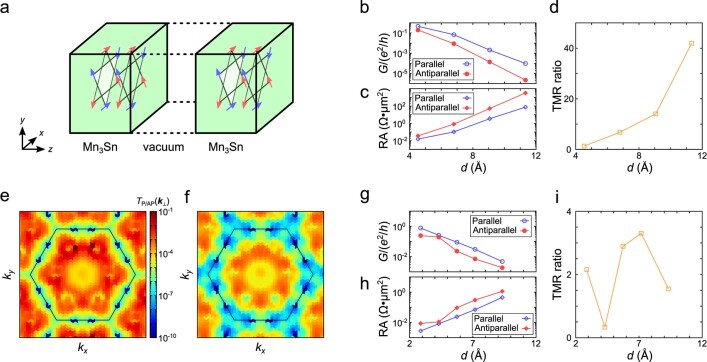
First-principles calculations of the tunneling magnetoresistance (TMR) effect with the vacuum barrier at the Fermi level. **a**, Schematics of the Mn_3_Sn/vacuum/Mn_3_Sn magnetic tunnel junction (MTJ). Blue and red arrows represent the magnetic moments carried by the Mn ions. **b**–**f**, Results for the Mn_3_Sn/vacuum/Mn_3_Sn MTJ. (**b**), (**c**) Vacuum thickness, *d*, dependence of the (**b**) total transmissions and (**c**) resistance-area products (RA) in the parallel and antiparallel configurations. (**d**) TMR ratio as a function of *d*. (**e**), (**f**) Transmissions resolved by the ***k***_⊥_-points for the (**e**) parallel and (**f**) antiparallel configurations at *d* = 9.062 Å, where the hexagons drawn by the black lines denote the first Brillouin zone. (**g**)–(**i**) Results for the Fe/vacuum/Fe MTJ. (**g**) Total transmissions and (**h**) RA values in the MTJ with the parallel and antiparallel configurations, and (**i**) TMR ratio with respect to *d*.

**Extended Data Fig. 4:**
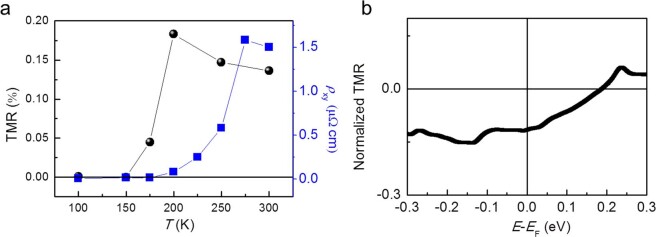
Interfacial Fermi level shift of Mn_3_Sn and resultant negative tunneling magnetoresistance. **a**, Evidence for interfacial Fermi level shift of interfacial Mn_3_Sn. The phase transition temperature from spin-spiral to noncollinear antichiral states of interfacial Mn_3_Sn (indicated by the TMR values (black circle, left)) is lower than the Mn_3_Sn films (indicated by the Hall resistivity *ρ*_*xy*_ (blue square, right)). **b**, Normalized TMR estimated as TMR = *τ*_bottom_ × *τ*_top_, assuming that octupole polarizations from bottom is *τ*_bottom_ and the one from top Mn_3_Sn is *τ*_top_, and there is 1 eV Fermi level shift between the top and bottom interfacial Mn_3_Sn. *E* − *E*_F_ is is the difference between band energy and the Fermi level.

To theoretically simulate the TMR effect from first principles, we use the PWCOND package in the QE package^57–59^ in which ballistic transport along the *z* direction is calculated by solving the scattering problem on the Bloch wave function obtained by DFT calculation^60^. Generally for TMR, it is important to have in-depth understanding of the barrier material^61–63^. On the other hand, before investigating the role of the barrier material it is crucial to determine whether there is any TMR effect in the all-antiferromagnetic tunnel junction. Thus, in our calculation, for simplicity we use Mn_3_Sn for the electrodes and the vacuum for the barrier. In practice, we calculate the tunnelling conductance in the Mn_3_Sn/vacuum/Mn_3_Sn MTJ. The MTJ in our calculations is made by stacking Mn_3_Sn along the *c* axis: the conducting path is perpendicular to the *ab* plane of Mn_3_Sn. The schematics of the MTJ are shown in Extended Data Fig. 3a. We calculated transmissions for both parallel and antiparallel configurations; the cluster magnetic octupole moments of the two electrodes point in the same and opposite directions in the parallel and antiparallel configurations, respectively.

First, we separate the entire MTJ system into three parts: left and the right leads consisting of the bulk Mn_3_Sn and the scattering region, comprising a pair of two monolayers of Mn_3_Sn and the vacuum region between. We performed the DFT calculation with QE for each of the three parts. We set the *k*-point mesh as 7 × 7 × 7 for the leads and 7 × 7 × 1 for the scattering region. In calculation of the scattering region, the constraint on magnetic moments was imposed to stabilize the magnetic structure. When we calculated the electronic structure of the scattering region in the antiparallel configuration, to smoothly connect the leads and the scattering region in the transmission calculation we treated the doubled scattering region, which consists of the original scattering region and its copy, attached to the original one with its magnetic configuration inverted. In this way we calculated the electronic structure of the Mn_3_Sn/vacuum/Mn_3_Sn/Mn_3_Sn/vacuum/Mn_3_Sn system. The doubled scattering region was cut in half and only the original was considered in transmission calculations.

Then, connecting the leads and scattering region, we calculated transmission. We set the *k*_⊥_ = (*k*_*x*_, *k*_*y*_) point mesh in the *xy* plane as 32 × 32. We obtained transmissions at each *k*_⊥_-point for the parallel/antiparallel configurations, *T*_P/AP_(*k*_⊥_); conductances for parallel/antiparallel configurations, *G*_P/AP_, were determined by the Landauer–Büttiker formula^64–67^, ${G}_{{\rm{P/AP}}}=({e}^{2}/h){\Sigma }_{{{\boldsymbol{k}}}_{\perp }}{T}_{{\rm{P/AP}}}({{\boldsymbol{k}}}_{\perp })$. The TMR ratio was calculated as (*G*_P_ − *G*_AP_)/*G*_AP_.

The vacuum thickness, *d*, dependence of the total transmission at the Fermi level for the parallel and antiparallel configurations, is shown in Extended Data Fig. 3b. We also plotted the resistance-area product (RA) for each configuration, which is the normalized resistance given as (RA) = *A*/*G*, where *A* is the cross-section area. We found that *G*_P_ was larger than *G*_AP_ in all cases of *d*, and both *G*_P_ and *G*_AP_ almost exponentially decayed with *d*, as shown in Extended Data Fig. 3b,c. We thus obtained the positive TMR ratio (Extended Data Fig. 3d). Transmissions resolved by *k*_⊥_-points are shown in Extended Data Fig. 3e,f, which indicates that transmission behaviour does differ between the parallel and antiparallel configurations.

For comparison with the Mn_3_Sn/vacuum/Mn_3_Sn MTJ, we also investigated the transmission properties of a ferromagnetic MTJ with the vacuum barrier, the Fe/vacuum/Fe system. In the same manner as for the Mn_3_Sn/vacuum/Mn_3_Sn MTJ, we calculated electronic structures without spin-orbit couplings of the leads composed by the bulk bcc-Fe with 8 × 8 × 8 *k*-mesh, and of the scattering region which has the vacuum sandwiched between a pair of four monolayers of Fe with 8 × 8 × 1 *k*-mesh. We performed the transmission calculation using 100 × 100 *k*_⊥_-point grids. Extended Data Fig. 3g,h shows the *d*-dependence of total transmissions and RA values at the Fermi level for both parallel and antiparallel arrangements. The TMR ratio with respect to *d* is plotted in Extended Data Fig. 3i, taking as large a value as that in the Mn_3_Sn/vacuum/Mn_3_Sn MTJ. The results should be sufficient to serve as a qualitative reference for the Mn_3_Sn/vacuum/Mn_3_Sn MTJ, whereas a small non-monotonic change in the TMR ratio was observed, which could converge by the calculation with higher accuracy.

The electronic states that dominate tunnelling transport correspond to electrons tunnelling in the normal direction to the interface, and thus the polarization of such states is important for discussion of tunnelling physics^68^. In fact, the tunnelling conductance of the Fe/MgO/Fe MTJ has the peak at *k*_⊥_ approximately 0, supporting this concept^61,62^. In a more complex system such as Mn_3_Sn, however, the states whose group velocity carries only normal incidence components exist not only at *k*_⊥_ approximately 0 but also at general *k* points^69^, and such states should largely contribute to tunnelling conductance following the concepts proposed by Slonczewski^68^. Extended Data Fig. 3e,f shows the in-plane momentum (*k*_*x*_, *k*_*y*_) dependence of transmission integrated over momentum along the tunnelling direction. Notably, our results clarify that transmission involves not only those states at *k*_⊥_ approximately 0, but also of the widely extended momentum region in the Brillouin zone. Given that the results in the figures are those projected to the in-plane momentum, tunnelling electrons arise not only from momentum *k*_⊥_ of approximately 0 related to normal incidence, but rather from entire region of the Brillouin zone. Thus, to qualitatively understand the mechanism of tunnelling conductance, we should focus not only on the states with *k*_⊥_ approximately 0 but rather use the measure reflecting contributions from the entire Brillouin zone. Given the fact that the antiferromagnetic state of Mn_3_Sn can be viewed as the ferroic order of cluster magnetic octupole, such a measure should be the summation of octupole polarization over the entire momentum space—the density of states projected onto octupole polarization.

### Effect of interfacial structures on transmission properties

We investigated the robustness of transmission properties against variation in interfacial structures in the Mn_3_Sn/vacuum/Mn_3_Sn MTJ. We examined two types of variation of the interface: interfacial disorder and lateral shift.

**Extended Data Fig. 5:**
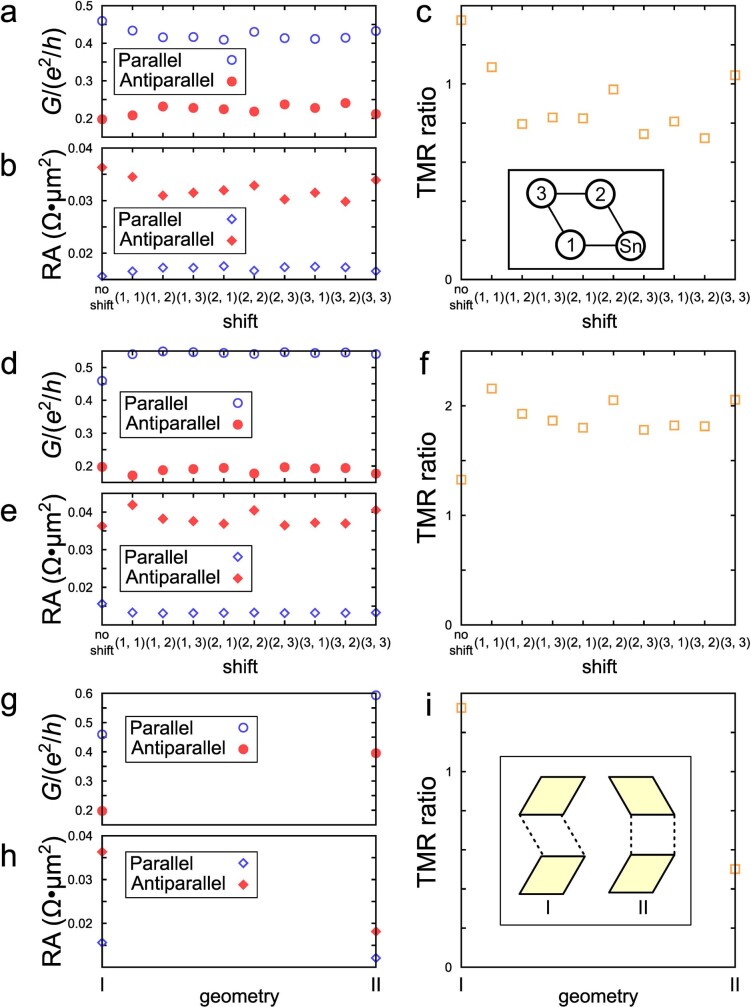
Effects of the interfacial disorder and the lateral shift on the tunneling magnetoresistance (TMR) of the Mn_3_Sn/vacuum/Mn_3_Sn magnetic tunnel junction (MTJ). The thickness of the barrier is 4.531 Å. **a,b,c**, Results for the MTJ with the atoms at the lower layer shifted upward and the upper layer downward. The pair of atoms shifted in the lower and upper layers is denoted as (*i*, *j*), where *i*, *j* = 1, 2, 3 correspond to the Mn-sites shown schematically in the inset in (c). (a) Total transmissions and (b) resistance-area products (RA) in the parallel and antiparallel configurations, and (c) the TMR ratio. **d,e,f**, Results for the MTJ with the atoms at the lower layer shifted downward and the upper layer upward. (d) Total transmissions and (e) RA in the parallel and antiparallel configurations, and (f) the TMR ratio. **g,h,i**, Results for the MTJ with two different geometries at the interface, which is schematically shown in the inset in (i) where the rhombuses indicate Mn_3_Sn. (g) Total transmissions and (h) RA in the parallel and antiparallel configurations, and (i) the TMR ratio.

First we studied the effect of disorders. We incorporated interfacial disorders into the Mn_3_Sn/vacuum/Mn_3_Sn MTJ by artificially moving some atoms facing the vacuum barriers; we shifted upwards one of the Mn atoms in the lower layer of Mn_3_Sn at the interface, and shifted downwards one of the Mn atoms in the upper layer of Mn_3_Sn. These atoms were shifted by 0.113 Å—that is, 2.5% of the *c* axis length of Mn_3_Sn, which does not qualitatively change the electronic and magnetic properties of the system. Extended Data Fig. 5a–c shows the results of calculations against the disorder with barrier thickness 4.531 Å. Whereas shifting of atoms decreased transmission in the parallel configuration and increased it in the antiparallel configuration, overall transmission properties did not change. We also examined the case in which atoms move inversely to the case above: the atom in the lower layer moved downwards whereas that in the upper layer moved upwards. The results of these calculations (Extended Data Fig. 5d–f) suggest that transmission properties did not qualitatively change in this case either. Whereas it is better to use a larger supercell for more precise evaluation of interfacial disorder, we expect that TMR properties may not largely change from those shown here.

Second, we examined the effect of lateral shift. Mn_3_Sn has two layers in a unit cell along the *c* axis—say, A and B. In the calculations whose results are shown in Fig. 3, layers A and B are at the interface, which we call geometry-I. Here we also consider the case in which two B-layers face each other (see the inset in Extended Data Fig. 5i), which we call geometry-II. We show the results of the calculations with geometries-I and II in Fig. 5g–i. These results indicate that the properties in tunnelling conductance do not largely change, whereas total transmissions in geometry-II take larger values than those in geometry-I. We note that for geometry-II we take the doubled unit cell both for the parallel and antiparallel configurations, due to its geometry. We confirmed that the finite TMR effect is observed for other lattice-matching configurations—that is, when two electrodes have differently oriented easy axes of cluster magnetic octupoles by 120° in geometry-I (Supplementary Fig. 4). We also examined the TMR effect by shifting the upper layer of Mn_3_Sn along the *a* axis by half of the lattice constant *a*, and confirmed that this shift qualitatively maintained the TMR effect (Supplementary Fig. 5).

### Bias-dependent TMR measurements

**Extended Data Fig. 6:**
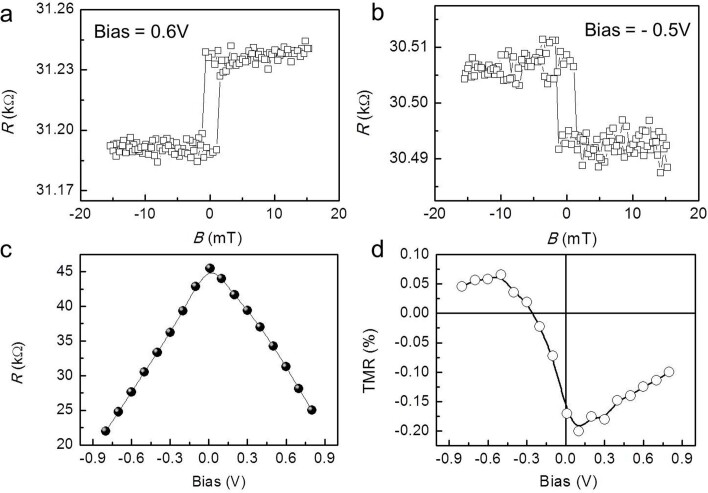
Bias dependent tunneling magnetoresistance of the Fe/MgO/Mn_3_Sn junction. **a,b**, Magnetic field dependence of the room temperature tunneling magnetoresistance of Fe(30)/MgO(3)/Mn_3_Sn(42) at the bias voltage of + 0.6 V (a) and − 0.5 V (b). The electric field and magnetic field are perpendicular (i.e. *E*⊥*B*) corresponding to Fig. 3c in the main text. The maximum magnetic field is 15 mT so that only Fe moments switch. Before scanning the small magnetic field, a large field of 1.5 T is applied to align the moment direction of Mn_3_Sn. **c**, Bias dependence of the tunneling resistance of Fe(30)/MgO(3)/Mn_3_Sn(42) from − 0.8 V to + 0.8 V. **d**, Bias dependence of the tunneling magnetoresistance ratio of Fe(30)/MgO(3)/Mn_3_Sn(42) from − 0.8 V to + 0.8 V.

The bias dependence of TMR can provide useful information about the energy dependence of polarization^41^. Thus, we performed TMR measurements as a function of bias voltage in the Fe/MgO/Mn_3_Sn tunnel junction for both the perpendicular (*E*⊥*B*) and parallel (E//B) configurations for the electric (*E*) and magnetic fields (*B*), corresponding to Fig. 3c,d, respectively. In the following, we show the experimental summary for the perpendicular (*E*⊥*B*) case as a representative example (Extended Data Fig. 6). Extended Data Fig. 6a,b shows the minor loops under bias voltage of +0.6 and −0.5 V, in which Fe moments are switched while Mn_3_Sn moments remain fixed.

**Extended Data Fig. 7:**
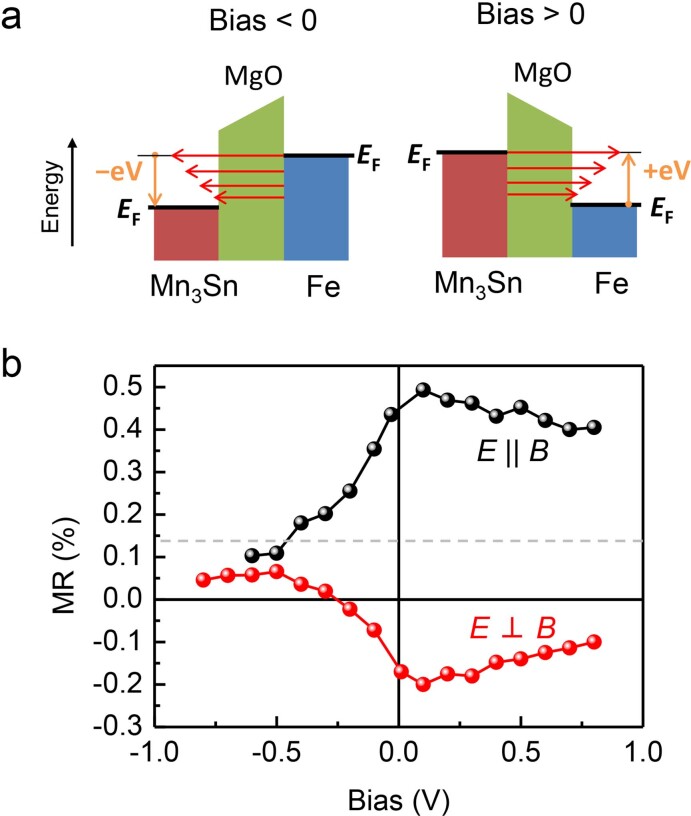
Bias dependent tunneling magnetoresistance of Fe/MgO/Mn_3_Sn tunnel junctions having the perpendicular or parallel configuration between the electric field and magnetic field. **a**, Schematic viewgraph indicating the relative position in energy for Mn_3_Sn and Fe. The bar indicates the simplified energy dependence of the density of states of the band in Mn_3_Sn and Fe. Tunneling conductance (red arrow) takes its maximum at the Fermi energy (*E*_F_). **b**, Bias dependence of the tunneling resistance of the MTJs having the parallel (*E//B*,V(30)/Fe(0.6)/MgO(3)/Mn_3_Sn(42)) and perpendicular (*E*⊥*B*, Fe(30)/MgO(3)/Mn_3_Sn(42)) configurations, measured between − 0.8 V to 0.8 V. The horizontal broken line is a guide to the eye.

The key observation is the symmetric bias dependence of the TMR ratio on the perpendicular and parallel configurations (Extended Data Fig. 7). This becomes clear if we consider a bias-independent term of the order of around 0.14 % (horizontal dashed line). According to our measurement configuration, the hot electron arises from Mn_3_Sn and thus MR should reflect the unoccupied DOS of Fe for positive bias (Extended Data Fig. 7a). In this regime, the spin polarization of Mn_3_Sn should be determined by the DOS of Mn_3_Sn in the vicinity of the Fermi level. Spin polarization around the Fermi level in Mn_3_Sn is anisotropic and can be characterized by the tensor, as discussed above. Thus, MR should have negative and positive signs for perpendicular and parallel configurations, respectively (Extended Data Fig. 7b). This explains the following observations of our experiment. Namely, after subtracting the constant background MR term of around 0.14%, we find that MR has magnitudes similar to the opposite signs for perpendicular and parallel configurations. Bias dependence must arise from the unoccupied DOS of Fe and should be similar to that observed in the Fe/MgO/Fe MTJ^5^.

On the other hand, negative bias should provide the hot electron from Fe to Mn_3_Sn. Thus, MR would be determined by the spin polarizations of unoccupied DOS for Mn_3_Sn and DOS for Fe in the vicinity of the Fermi level. As compared with the positive bias regime, MR in the negative bias is strongly bias dependent (Extended Data Fig. 7b). This would be due to the bias voltage dependence of the anisotropic spin polarization in Mn_3_Sn. Interestingly, the MRs for both perpendicular and parallel MTJs converge at around 0.1% at bias V below −0.5. Most probably, this is a result of isotropic spin polarization of Mn_3_Sn from spin canting.

### Measurement of the thickness dependence of TMR

**Extended Data Fig. 8:**
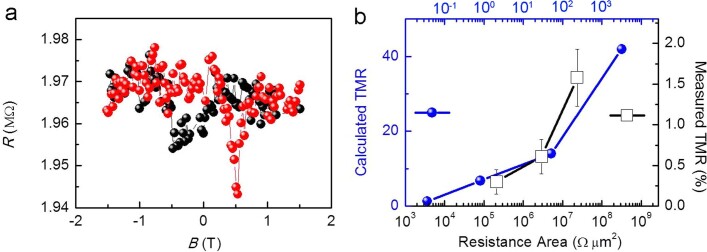
Tunneling magnetoresistance of Mn_3_Sn/MgO/Mn_3_Sn stacks with different MgO thickness. **a**, Field dependence of the tunneling magnetoresistance of Mn_3_Sn(12)/MgO(3.3)/Mn_3_Sn(42) at room temperature. The magnetic field and electric current are both applied perpendicular to the Mn_3_Sn plane. **b**, Comparison between experimental tunneling magnetoresistance of Mn_3_Sn/MgO/Mn_3_Sn and numerically calculated tunneling magnetoresistance of Mn_3_Sn/vacuum/Mn_3_Sn as a function of resistance area.

**Extended Data Fig. 9:**
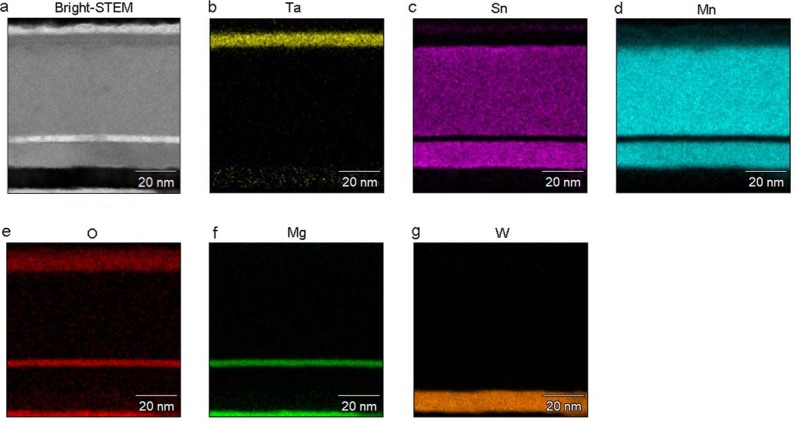
Energy dispersive X-ray spectroscopy mapping of Mn_3_Sn/MgO/Mn_3_Sn stacks. a-g, Elemental mappings for the area of Bright-STEM images (a) are shown in (b) Ta (yellow), (c) Sn (purple), (d) Mn (blue), (e) O (red), (f) Mg (green), and (g) W (orange). The white scale bars in (a-g) represent a length of 20 nm. Smooth W/Mn_3_Sn and Mn_3_Sn/MgO interfaces are clearly observed.

**Extended Data Fig. 10:**
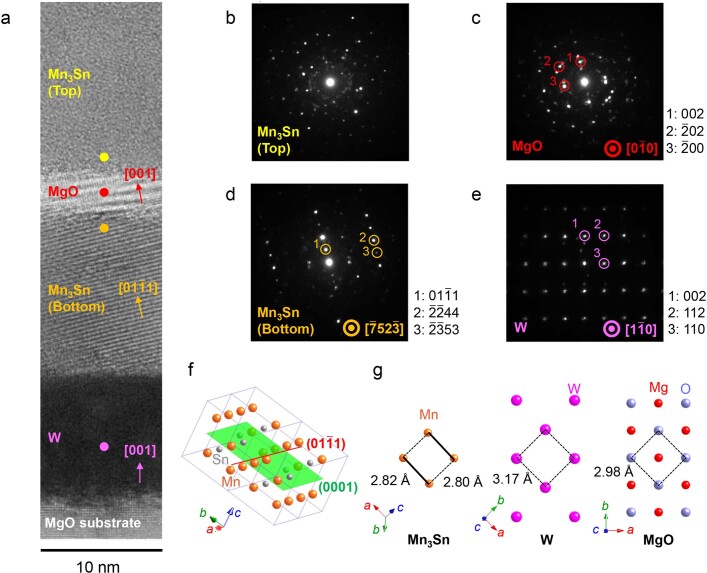
Transmission electron microscopy (TEM) images for the Mn_3_Sn/MgO/Mn_3_Sn stack. **a**. Cross-sectional TEM image of the heterostructure of the AFMTJ, MgO(001) substrate/W/Mn_3_Sn/MgO/Mn_3_Sn/Ta. **b—e**. Nano-beam electron diffraction patterns of the Mn_3_Sn (top) (b), MgO (c), Mn_3_Sn (bottom) (d), and W (e) layers. Measurement spots with *ϕ* 1 nm diameter are indicated as filled circles in the panel (a). Indexed spots are indicated by open circles and corresponding lattice planes are described next to the images. **f**. Representative crystal structure of Mn_3_Sn in the Mn_3_Sn bottom layer derived from the diffraction in the panel (d). Transparent light green plane and red plane indicate the plane parallel to the kagome (0001) plane and $(01\bar{1}1)$ plane, respectively. The unit cell of Mn_3_Sn is denoted by a solid blue line. The viewpoint is the same as in the panels (a) and (d). **g**. Schematics of the atomic arrangement in the $(01\bar{1}0)$ plane of Mn_3_Sn and (001) plane of W and MgO.

The TMR in Mn_3_Sn/MgO/Mn_3_Sn MTJ was measured by variation in MgO thickness. When the barrier became thicker, TMR value increased, consistent with the theoretical calculations shown in Extended Data Fig. 2. We also performed a comparison between experiments and calculations, as shown in Extended Data Fig. 8. Whereas these calculations use the vacuum barrier, we set the *x* axis to be the resistance area rather than MgO thickness itself, for clarity.

## Online content

Any methods, additional references, Nature Portfolio reporting summaries, source data, extended data, supplementary information, acknowledgements, peer review information; details of author contributions and competing interests; and statements of data and code availability are available at 10.1038/s41586-022-05463-w.

### Supplementary information

Supplementary Information Supplementary Figs. 1–5 and references.

## Data Availability

The data that support the findings of this study are available from the corresponding authors on reasonable request.

## Source data

Source Data Fig. 1
Source Data Fig. 2
Source Data Fig. 3
