# Supplementary material for: Octupole-driven magnetoresistance in an antiferromagnetic tunnel junction
Source: Nature. 2023 Jan 18;613(7944):490–5. doi: 10.1038/s41586-022-05463-w (PMC9849134; doi:10.1038/s41586-022-05463-w)
Supplement: Supplementary file 1 — Supplementary Figs. 1–5 and references. [file 41586_2022_5463_MOESM1_ESM.pdf]

nature portfolio

https://doi.org/10.1038/s41586-022-05463-w

Supplementary information

Octupole-driven magnetoresistance in an antiferromagnetic tunnel junction

In the format provided by the authors and unedited

Supplementary Information for 'Octupole-driven magnetoresistance in an antiferromagnetic tunnel junction'

Xianzhe Chen[1,2†], Tomoya Higo[1,2,3†], Katsuhiro Tanaka[4†], Takuya Nomoto[4, 5], Hanshen Tsai[1,2], Hiroshi Idzuchi[1,2], Masanobu Shiga[2], Shoya Sakamoto[2], Ryoya Ando[2], Hidetoshi Kosaki[2], Takumi Matsuo[1], Daisuke Nishio-Hamane[2], Ryotaro Arita[3,4,6], Shinji Miwa[2,3,7], and Satoru Nakatsuji[1-3,7*]

[1]*Department of Physics, University of Tokyo, Bunkyo-ku, Tokyo 113-0033, Japan*
[2]*Institute for Solid State Physics, University of Tokyo, Kashiwa, Chiba 277-8581, Japan*
[3]*CREST, Japan Science and Technology Agency, Kawaguchi, Saitama 332-0012, Japan*
[4]*Department of Applied Physics, University of Tokyo, Bunkyo-ku, Tokyo 113-8656, Japan*
[5]*PRESTO, Japan Science and Technology Agency, Kawaguchi, Saitama 332-0012, Japan*
[6]*RIKEN, Center for Emergent Matter Science (CEMS), Saitama 351-0198, Japan*
[7]*Trans-scale Quantum Science Institute, University of Tokyo, Bunkyo-ku, Tokyo 113-0033, Japan*

TMR calculations with *ab*-plane of Mn_3Sn perpendicular and parallel to the conducting path
For the numerical results for $\mathrm{Mn_3Sn}$/vacuum/$\mathrm{Mn_3Sn}$ MTJ shown in Extended Data Figs. 3 and 5, the ab-plane of $\mathrm{Mn_3Sn}$ is perpendicular to the conducting path. In addition to this configuration, we have also performed the TMR calculation with $\mathrm{Mn_3Sn}$/vacuum/$\mathrm{Mn_3Sn}$, where the ab-plane of $\mathrm{Mn_3Sn}$ is parallel to the tunneling direction. The barrier thickness dependence of the transmission properties are shown in Supplementary Fig. 1. We note that in the TMR calculation where the ab-plane of $\mathrm{Mn_3Sn}$ is parallel to the conducting path, the scattering region of the parallel magnetic configuration has a weak ferromagnetic moment $\sim 0.03\ \mu_\mathrm{B}$/Mn on average due to canting. Notably, this value is still too small to account for the observed TMR in the all antiferromagnetic MTJ by the mechanism based on the spin polarization. We also remark that due to the numerical limitations the size of in-plane component of the cell is limited to one unit of the in-plane lattice constant a when the kagome-plane is parallel to the tunneling direction. Hence, the results could give only qualitative information and the value itself should be examined carefully.

Anisotropic spin current in Mn_3Sn
In Mn_3Sn, the spin conductivity tensor is anisotropic[1], as illustrated in Supplementary Fig. 2. When we take the right-handed Cartesian coordinate and the kagome plane on the ij plane, σ^j_{ii} is negative (left) and σ^i_{ii} is positive (right). Namely, when the electric (E) and magnetic fields (B) are perpendicular (left), the spin current flows towards the opposite direction to the electric field E while having the spin polarization direction along the same direction as B (left). In the parallel situation ($E \mathbin{/\!/} B$, right), the longitudinal spin current flows along the same direction as E having the spin polarization direction along $E \mathbin{/\!/} B$ (right). Here, the relative angle between the kagome lattice and the coordinate $\{ij\}$ is irrelevant, as long as the spin-orbit coupling is small as in Mn_3Sn. Thus, this behavior should be observed even in a polycrystalline sample. The discovery of the anisotropic, longitudinal spin-polarized current for the first time in an antiferromagnet is

significant as it allows us to employ not only the TMR, but the STT-control of the propagation of the antiferromagnetic domain walls, enabling the design of the antiferromagnetic STT-MRAM.

Inelastic electron tunneling spectrum

Inelastic electron tunneling (IET) spectrum has been recognized as an efficient way to probe the role of the electron-magnon scattering as well as the properties of MTJs [2]. We have performed the measurements of the bias dependence at different temperatures for Mn_3Sn/MgO/Mn_3Sn and obtained the IET spectrum (d^2I/dV^2) as a function of the bias voltage V. The I-V curve systematically changes its slope on cooling (Supplementary Fig. 3a). Based on this, the IET spectra are obtained from 300 K to 50 K, as shown in Supplementary Fig. 3b.

Most notable is the change in the IET spectrum seen between 300 K and 200 K. This is in contrast with the data for the temperature range between 200 K and 50 K. Below 200 K, the IET spectra are weakly sharpening around zero bias voltage on cooling, and below 100 K the results overlap on top of each other. This indicates that the large spectrum change seen between 300 K and 200 K is not due to thermal broadening.

Instead, this originates most likely from the magnetic transition between the antichiral phase and the spiral phase that takes place between 300 K and 200 K. The antichiral phase hosts the ferroic order of the cluster magnetic octupole and may produce TMR in Mn_3Sn/MgO/Mn_3Sn MTJs as we observe at 300 K. On the other hand, in the spiral phase seen below $T = 200$ K, there is no longer the ferroic order of the octupole and thus no TMR is expected.

The antichiral phase becomes unstable on cooling in Mn_3Sn but remain intact at low temperatures in Mn_3Ge. Recent inelastic neutron scattering experiments find that the antichiral phase in both Mn_3Sn and Mn_3Ge have the magnetic excitations localized in the energy range between 50 meV and 80 meV [3]. In comparison, it has been shown that the spiral phase harbors the magnetic excitations in a wider energy range above 40 meV and even beyond 100 meV [4]. One of the reasons behind the larger excitation energy in the spiral phase is the increase in the magnetic moment size from 2.3 μ_B/Mn in the antichiral phase to 3.1 μ_B/Mn in the spiral phase.

Therefore, it is conceivable that the evolution in the IET spectra below and around 100 meV on cooling from 300 K and 200 K should be related to the change in the magnetic excitations due to the magnetic phase transition. It is likely that the magnon-electron scattering in the same region of the bias voltage may play an important role in the tunnelling conductance and thus in TMR.

References

1. Železný, J., Zhang, Y., Felser, C. & Yan, B. Spin-polarized current in noncollinear antiferromagnets. *Phys. Rev. Lett.* **119**, 187204 (2017).

2. Ando, Y., Murai, J., Kubota, H. & Miyazaki, T. Magnon-assisted inelastic excitation spectra of a ferromagnetic tunnel junction. *Journal of Applied Physics* **87**, 5209–5211 (2000).

3. Chen, Y. *et al.* Antichiral spin order, its soft modes, and their hybridization with phonons in the topological semimetal Mn_3Ge. *Phys. Rev. B* **102**, 054403 (2020). URL `https://link.aps.org/doi/10.1103/PhysRevB.102.054403`.

4. Park, P. *et al.* Magnetic excitations in non-collinear antiferromagnetic Weyl semimetal Mn_3Sn. *npj Quantum Materials* **3**, 1–8 (2018).

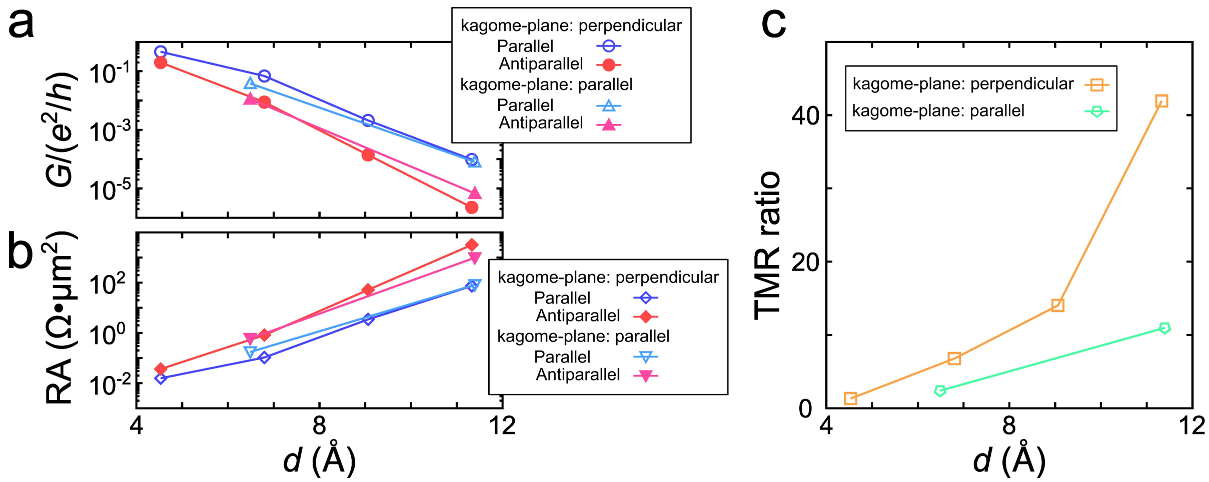

Supplementary Figure 1: Numerical calculation results of the tunneling magnetoresistance for the Mn_3Sn/vacuum/Mn_3Sn magnetic tunnel junctions where the *ab*-plane of Mn_3Sn is parallel and perpendicular to the conducting path. a,b,c, Barrier thickness d dependence of (a) the total transmissions, (b) resistance-area products, and (c) the absolute values of the TMR ratios.

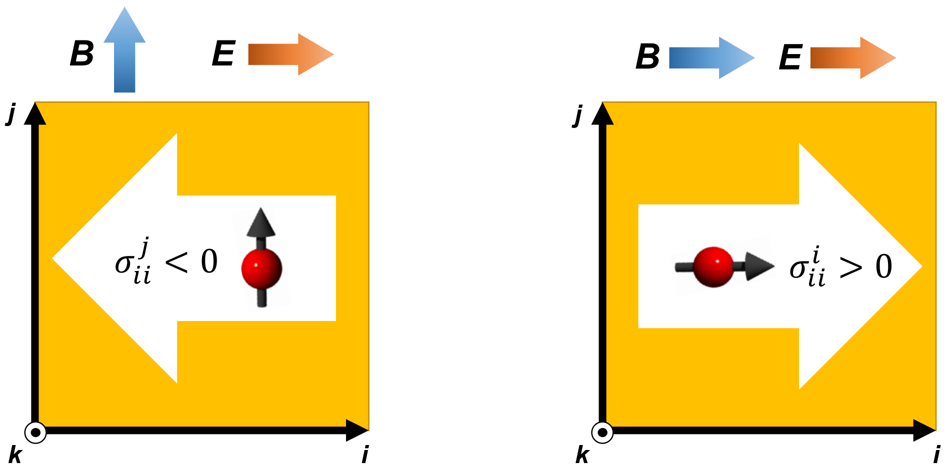

Supplementary Figure 2: Schematic illustration of the anisotropic spin conductivity tensor σ_{ij}^{k}. Black arrows denote the spin magnetic moments of conduction electrons (red spheres). For detailed explanation, see the text in Sec. "Anisotropic spin current in Mn_3Sn" of Supplementary Information.

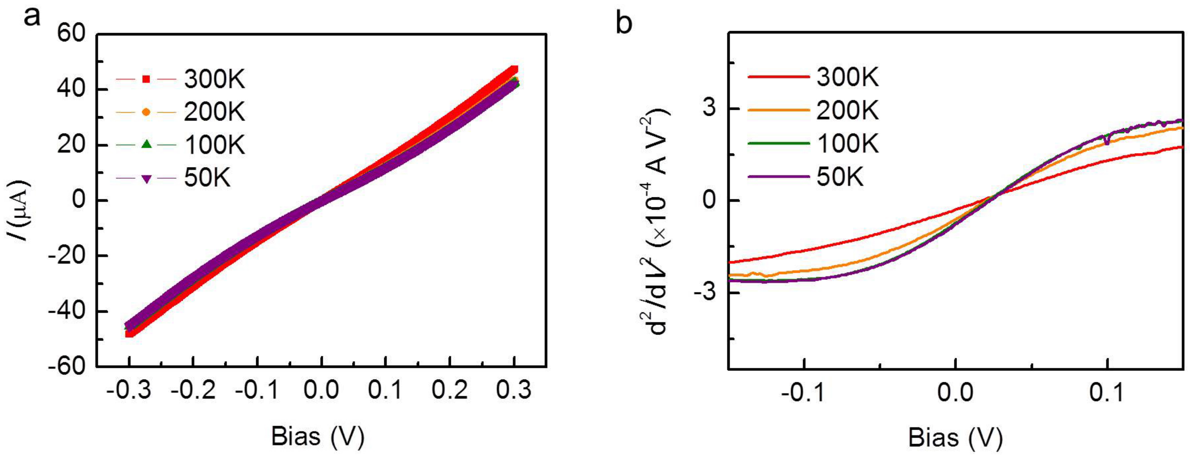

Supplementary Figure 3: Inelastic electron tunneling spectrum of Mn_3Sn/MgO/Mn_3Sn tunnel junction at different temperatures. **a**, Bias voltage dependence of the tunneling current, **b**, d^2I/dV^2 recorded as a function of bias voltage at various temperatures of 300K, 200 K, 100 K, and 50 K.

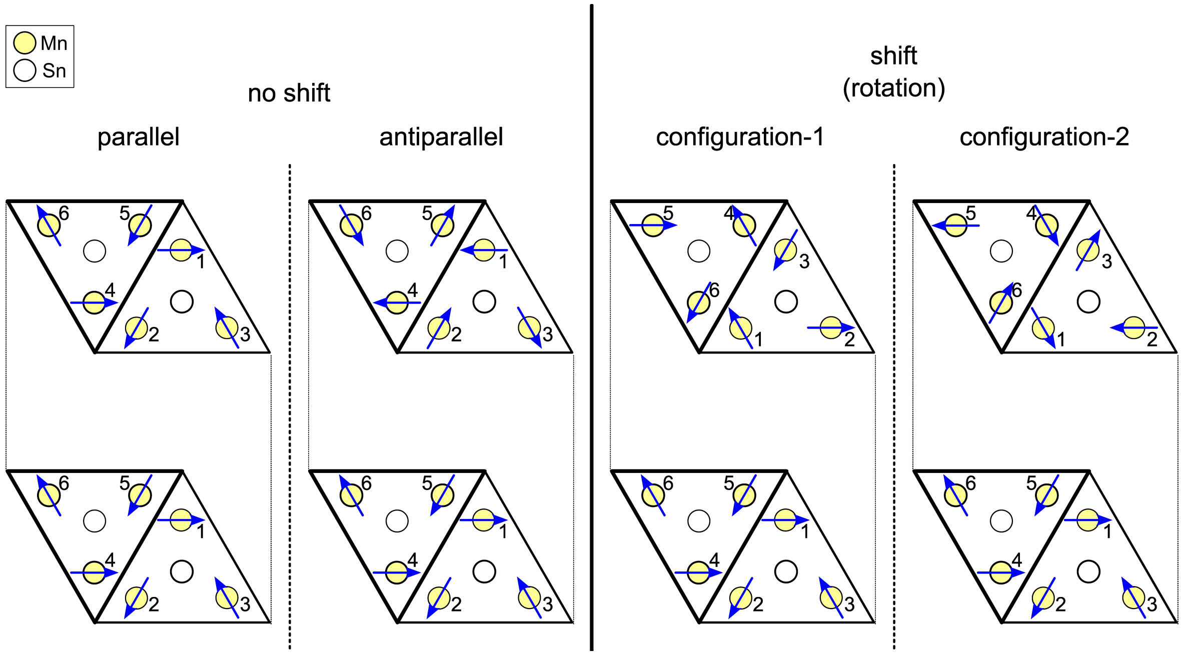

Supplementary Figure 4: Schematics of the atomic and magnetic structures of the Mn_3Sn/vacuum/Mn_3Sn tunnel junctions without shift (left) and with 'lateral shift' (right). These represent two lattice matching configurations where Mn atoms are on top of each other and when we consider the case where A and B are facing each other. Here A and B refer to two ab-plane layers of the Mn_3Sn unit cell. Numbers (1–6) distinguish the different Mn ions. Arrows denote the magnetic structures. Top and bottom rhombuses represent the electrodes on both sides. The thickness of the vacuum barrier is set as 4.531 Å. The "configuration-1" and "configuration-2" are realized via rotating by $120°$ the parallel and antiparallel configurations of the original structures of the top electrode, respectively. We calculate the transmissions for these configurations with 32×32 $\boldsymbol{k}$-point grids. The TMR ratio, given by $(G_2 - G_1)/G_1$, is found to be 0.37 (37 %), where $G_{1/2}$ is the conductance for the configuration-1/2.

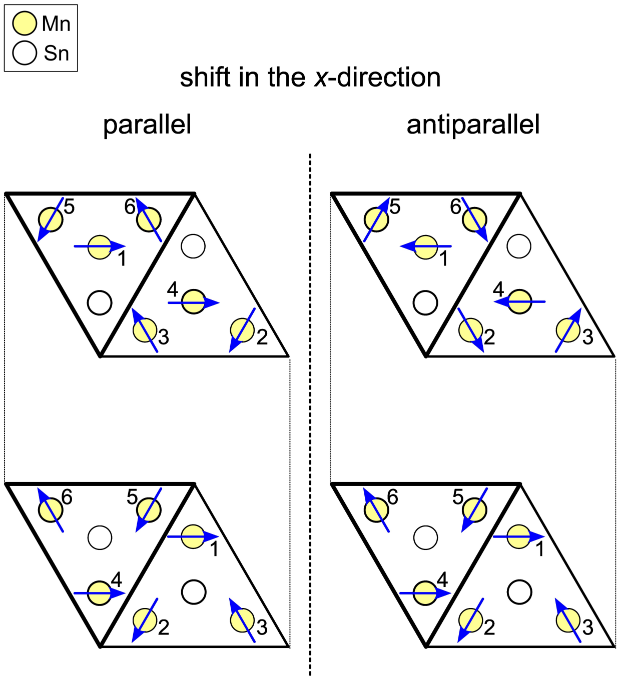

Supplementary Figure 5: Schematic picture of the crystal and magnetic structures of the Mn_3Sn/vacuum/Mn_3Sn tunnel junctions with the lateral shift. The lateral shift is realized by shifting the atoms of the top in the x-direction by half of the lattice constant a. We distinguish the different Mn atoms by numbers 1–6, and arrows represent the magnetic structures. We set the thickness of the barrier as 4.531 Å. We perform the transmission calculation with 32×32 $\boldsymbol{k}$-point grids. The TMR ratio is 0.92 (92%), which has the same sign as and a similar magnitude to the one without shift, 1.3 (130%).
